# Clinical characteristics and outcomes of immunocompromised critically ill patients with cytomegalovirus end-organ disease: a multicenter retrospective cohort study

**DOI:** 10.1186/s13054-024-05029-4

**Published:** 2024-07-16

**Authors:** Sara Fernández, Ignacio Grafia, Olivier Peyrony, Emmanuel Canet, Clara Vigneron, Clément Monet, Nahéma Issa, Maxens Decavele, Anne-Sophie Moreau, Alexandre Lautrette, Guillaume Lacave, Guillaume Morel, Cyril Cadoz, Laurent Argaud, Liran Statlender, Karam Azem, Jean-Pierre Quenot, Olivier Lesieur, Javier Fernández, Marta Farrero, Mª Ángeles Marcos, Virgine Lemiale, Pedro Castro, Élie Azoulay

**Affiliations:** 1 Medical Intensive Care Unit, Hospital Clínic of Barcelona, Barcelona, Spain; 2 Medical Intensive Care Unit, Hôpital Saint-Louis, Assistance Publique-Hôpitaux de Paris (AP-HP), Paris, France; 3 Department of Medical Oncology, IDIBAPS, Hospital Clinic of Barcelona, Barcelona, Spain; 4 Emergency Department, Hôpital Saint-Louis, Assistance Publique-Hôpitaux de Paris (AP-HP), Paris, France; 5 Department of Infectious Diseases, Hospital Clinic of Barcelona, Barcelona, Spain; 6 Service de Médecine Intensive Réanimation, CHU de Nantes, Université de Nantes, Nantes, France; 7 Médecine Intensive et Réanimation, Hôpital Cochin, Assistance Publique-Hôpitaux de Paris (AP-HP), Paris, France; 8 Anesthesia and Critical Care Department, St-Eloi Hospital, University of Montpellier, PhyMedExp, INSERM U1046, CNRS, Montpellier, France; 9 Medical Intensive Care Unit, Hôpital Saint André, CHU Bordeaux, Bordeaux, France; 10 Medical Intensive Care Unit (Department R3S), Pitié-Salpêtrière University Hospital, Sorbonne University Hospitals, Assistance Publique-Hôpitaux de Paris (AP-HP), Paris, France; 11 INSERM Research Unit UMRS1158, Experimental and Clinical Respiratory Neurophysiology, Sorbonne University, Paris, France; 12 Medical Intensive Care Unit, CHU Lille, Lille, France; 13 Medical Intensive Care Unit, Jean Perrin Oncology Institut and Montpied Teaching Hospital, Clermont-Ferrand, France; 14 Medical-Surgical Intensive Care Unit, Versailles Hospital Center, Le Chesnay, France; 15 Hematology Department, Institut Paoli-Calmettes, Marseille, France; 16 Réanimation Polyvalente, CHR Metz-Thionville Hôpital de Mercy, Metz, France; 17 Medical Intensive Care Unit, Hôpital Edouard Herriot, Hospices Civils de Lyon, Lyon, France; 18 General Intensive Care Unit, Beilinson Hospital, Rabin Medical Center, Petah Tikva, Israel; 19 School of Medicine, Tel Aviv University, Tel Aviv, Israel; 20 Anesthesiology Department, Beilinson Hospital, Rabin Medical Center, Petah Tikva, Israel; 21 Deparment of Intensive Care, Burgundy University Hospital, Dijon, France; 22 Intensive Care Unit, La Rochelle General Hospital, La Rochelle, France; 23 Liver Intensive Care Unit, Hospital Clinic of Barcelona, Barcelona, Spain; 24 Institut d’Investigacions Biomèdiques August Pi i Sunyer (IDIBAPS), University of Barcelona, Barcelona, Spain; 25 EF-Clif, Barcelona, Spain; 26 Heart Failure Unit, Hospital Clinic of Barcelona, Barcelona, Spain; 27 Microbiology Department, Hospital Clinic of Barcelona, University of Barcelona, Barcelona, Spain; 28 Centro de Investigación Biomédica en Red de Enfermedades Infecciosas, Instituto de Salud Carlos III, Madrid, Spain

**Keywords:** Cytomegalovirus, Intensive care, Immunocompromised host, Transplantation, Hematologic malignancy

## Abstract

**Background:**

Cytomegalovirus (CMV) infection in patients with cellular immune deficiencies is associated with significant morbidity and mortality. However, data on CMV end-organ disease (CMV-EOD) in critically ill, immunocompromised patients are scarce. Our objective here was to describe the clinical characteristics and outcomes of CMV-EOD in this population.

**Methods:**

We conducted a multicenter, international, retrospective, observational study in adults who had CMV-EOD and were admitted to any of 18 intensive care units (ICUs) in France, Israel, and Spain in January 2010–December 2021. Patients with AIDS were excluded. We collected the clinical characteristics and outcomes of each patient. Survivors and non-survivors were compared, and multivariate analysis was performed to identify risk factors for hospital mortality.

**Results:**

We studied 185 patients, including 80 (43.2%) with hematologic malignancies, 55 (29.7%) with solid organ transplantation, 31 (16.8%) on immunosuppressants, 16 (8.6%) with solid malignancies, and 3 (1.6%) with primary immunodeficiencies. The most common CMV-EOD was pneumonia (n = 115, [62.2%] including 55 [47.8%] with a respiratory co-pathogen), followed by CMV gastrointestinal disease (n = 64 [34.6%]). More than one organ was involved in 16 (8.8%) patients. Histopathological evidence was obtained for 10/115 (8.7%) patients with pneumonia and 43/64 (67.2%) with GI disease. Other opportunistic infections were diagnosed in 69 (37.3%) patients. Hospital mortality was 61.4% overall and was significantly higher in the group with hematologic malignancies (75% vs. 51%, *P* = 0.001). Factors independently associated with higher hospital mortality were hematologic malignancy with active graft-versus-host disease (OR 5.02; 95% CI 1.15–27.30), CMV pneumonia (OR 2.57; 95% CI 1.13–6.03), lymphocytes < 0.30 × 10^9^/L at diagnosis of CMV-EOD (OR 2.40; 95% CI 1.05–5.69), worse SOFA score at ICU admission (OR 1.18; 95% CI 1.04–1.35), and older age (OR 1.04; 95% CI 1.01–1.07).

**Conclusions:**

Mortality was high in critically ill, immunocompromised patients with CMV-EOD and varied considerably with the cause of immunodeficiency and organ involved by CMV. Three of the four independent risk factors identified here are also known to be associated with higher mortality in the absence of CMV-EOD. CMV pneumonia was rarely proven by histopathology and was the most severe CMV-EOD.

**Supplementary Information:**

The online version contains supplementary material available at 10.1186/s13054-024-05029-4.

## Background

Cytomegalovirus (CMV) is among the most prevalent causes of opportunistic infection (OI) in patients with impaired cellular immunity and is particularly common in recipients of allogeneic hematopoietic stem cell transplants (HSCT) or solid organ transplants (SOT) [1, 2]. However, susceptibility to CMV infection is increasing in non-transplanted patients due to the expanding use of high-dose corticosteroid therapy and introduction of new immunosuppressive drugs [3, 4]. Reactivation of dormant virus is the most common mechanism [1, 2]. CMV end-organ disease (CMV-EOD) is invasion of one or more organs by the virus, which may induce organ failures requiring admission to the intensive care unit (ICU).

CMV reactivation has been reported in up to a third of seropositive immunocompetent patients in the ICU, with sepsis and mechanical ventilation being associated with the highest rates. Adverse outcomes associated with CMV reactivation included longer invasive mechanical ventilation (MV) duration, longer ICU stay, and higher mortality [5–8]. In the critically ill, the risk of CMV-EOD and organ dysfunction in the event of CMV reactivation may be higher than in other patients and the contribution of CMV to mortality therefore greater [9]. The risks would be expected to be highest in immunocompromised patients. In addition, CMV can modify immune-system responses in various ways, thereby inducing adverse effects such as an increased risk of OIs [10, 11]. However, data on CMV-EOD in critically ill, immunocompromised patients are scarce. Such data are needed to identify those patients at highest risk thereby potentially improving the early diagnosis and decreasing CMV-associated morbidity and mortality.

The objective of this multicenter international retrospective observational study was to describe the clinical characteristics and outcomes of critically ill, immunocompromised patients with CMV-EOD.

## Methods

### Study design and population

We conducted a multicenter, retrospective, observational study in 18 ICUs in France, Israel, and Spain. The study was approved by the appropriate French ethics committee (*Societé de Réanimation de Langue Française*, CE SRLF 22-036, 06/07/2022), Spanish ethics committee (*Comité de Ética de la Investigación con medicamentos [CEim] del Hospital Clínic de Barcelona*, HCB/2022/0333, 31/03/2022), and Israeli ethics committee (Rabin Medical Center Institutional review board [IRB], RMC-0661-22, 16/05/2022). All three ethics committees waived the need for patient informed consent, in compliance with local legislation on retrospective analyses of de-identified health data.

Adults (≥ 18 years) who had probable or proven active CMV-EOD, immunosuppression, and ICU admission between January 2010 and December 2021 were identified in each participating ICU based on coded diagnoses of CMV infection or disease or microbiological data. We did not include immunocompetent patients or patients with acquired immunodeficiency syndrome. Patients with any of the following causes of immunodeficiency were eligible: hematologic malignancy (with or without HSCT for any reason), SOT, solid malignancy other than localized skin cancer and either active or in remission for less than 5 years, primary immune deficiency, and drug-induced immunosuppression defined as corticosteroid therapy in a dose > 0.5 mg/Kg/day and/or one or more other immunosuppressant drugs for longer than 30 days. CMV reactivation was considered when a positive CMV DNA determined by quantitative polymerase chain reaction (qPCR) in blood/plasma or any other body fluid was found, according to the specific detection threshold used by the CMV viral load assays at each center. CMV-EOD was defined as tissue-invasive CMV infection directly responsible for organ damage demonstrated by the presence of clinical signs and symptoms specific of the organ involved plus detection of CMV in tissue by histopathology, immunohistochemistry, or DNA hybridization techniques, virus isolation, or rapid culture. CMV retinitis was defined by the presence of typical ophthalmological signs judged by an experienced ophthalmologist. Patients with clinical signs and symptoms specific of the organ involved with detection of CMV by viral isolation, rapid culture or quantitative CMV DNA by PCR in bronchoalveolar (BAL) or cerebrospinal fluid (CSF) were included as probable pneumonia or probable encephalitis respectively. Patients with clinical signs and symptoms of GI disease, with macroscopic mucosal lesions and high CMV DNA levels detected by quantitative CMV DNA by PCR in gastrointestinal tissue samples comparing to blood viral load but without histopathological changes, in the absence of other possible diagnosis, were also considered as possible GI disease. Disseminated CMV disease was defined as CMV disease involving more than one organ, following the previous definitions. A positive blood CMV qPCR together with the presence of symptoms and signs, but without an additional test to detect CMV on tissue biopsies or body fluid samples were not considered enough for the diagnosis of CMV-EOD or disseminated CMV disease.

The general practice for the treatment of CMV-EOD was to start intravenous ganciclovir as the first drug of choice, following the international guidelines recommendations [12, 13]. In those patients in whom it was not possible to administer ganciclovir due to severe cytopenia or in whom ganciclovir did not achieve clinical outcomes or virological clearance, foscarnet was used as an alternative second-line agent for treatment.

### Data collection

The variables collected were designed specifically for this study and were collected retrospectively once the patients were identified. A local investigator in each participating ICU used standardized forms to abstract the following data from the medical records of each patient: baseline characteristics at ICU admission, including the cause of immunosuppression before ICU admission; Sequential Organ Failure Assessment (SOFA) score [14] at ICU admission as a marker of acute illness severity; CMV DNA loads in blood and other body fluids measured by qPCR and reported as World Health Organization standard IU/mL [15]; date of CMV-EOD diagnosis defined as the date of first CMV detection by histopathological examination, qPCR, or viral culture in fluid and/or tissue samples; organ or organs affected by CMV disease; clinical and laboratory data at diagnosis of CMV-EOD; and anti-CMV drugs administered. Blood CMV qPCR was monitored once or twice a week in transplant patients (both SOT and HSCT) according to the specific local protocol of each center. Patients with CMV-EOD were monitored for resolution of clinical signs and symptoms related to CMV-affected organs, along with weekly monitoring of blood CMV qPCR to verify virologic clearance, until CMV DNAemia declined to undetectable levels or below a predefined viral load threshold or until they were discharged from the ICU.

Other infections, including OIs, were recorded and classified as either concomitant (diagnosed within 48 h) with the diagnosis of CMV-EOD or developed during the ICU stay. Finally, organ-support interventions, ICU mortality, hospital mortality and day-90 mortality were recorded.

### Statistical analysis

Continuous variables were described as median [interquartile range] and categorical variables as number (%). To compare hospital survivors and non-survivors, we applied the nonparametric Mann–Whitney test for continuous variables and the chi-square test or Fisher exact test, depending on sample size, for categorical variables. Comparison between median CMV viral load levels in BAL fluid or CFS and blood samples in patients with probable pneumonia or encephalitis, respectively, was performed using the Wilcoxon signed-rank test [16].

A multivariable logistic regression model was built to identify risk factors for hospital mortality. Variables associated with hospital mortality at *P* values smaller than 0.05 by univariable analysis, together with clinically relevant variables, were entered into the model: age, SOFA score, type of immunosuppression, CMV-EOD, lymphocytes and platelet count at CMV-EOD diagnosis and previous treatment with corticosteroids. Variables related with organ support (renal replacement therapy, mechanical ventilation and use of vasopressors) were not used in the model as they were considered redundant with the SOFA score. Likewise, previous chemotherapy was removed from the model because there was a collinearity with the type of immunosuppression. Variables that were not available at ICU admission (such as aspergillosis or other opportunistic infection during ICU stay) were also removed from the model.

The patients were divided into groups based on the cause of immune deficiency and on the organ or organs involved by CMV. Day-90 survival was compared across patient groups by plotting Kaplan–Meier curves then applying the log-rank test. All *P* values were two-sided, and values of 0.05 or less were considered statistically significant. The data were analyzed using the R program (R Core Team, 2013; https://www.r-project.org).

## Results

### Study population

**Fig. 1:**
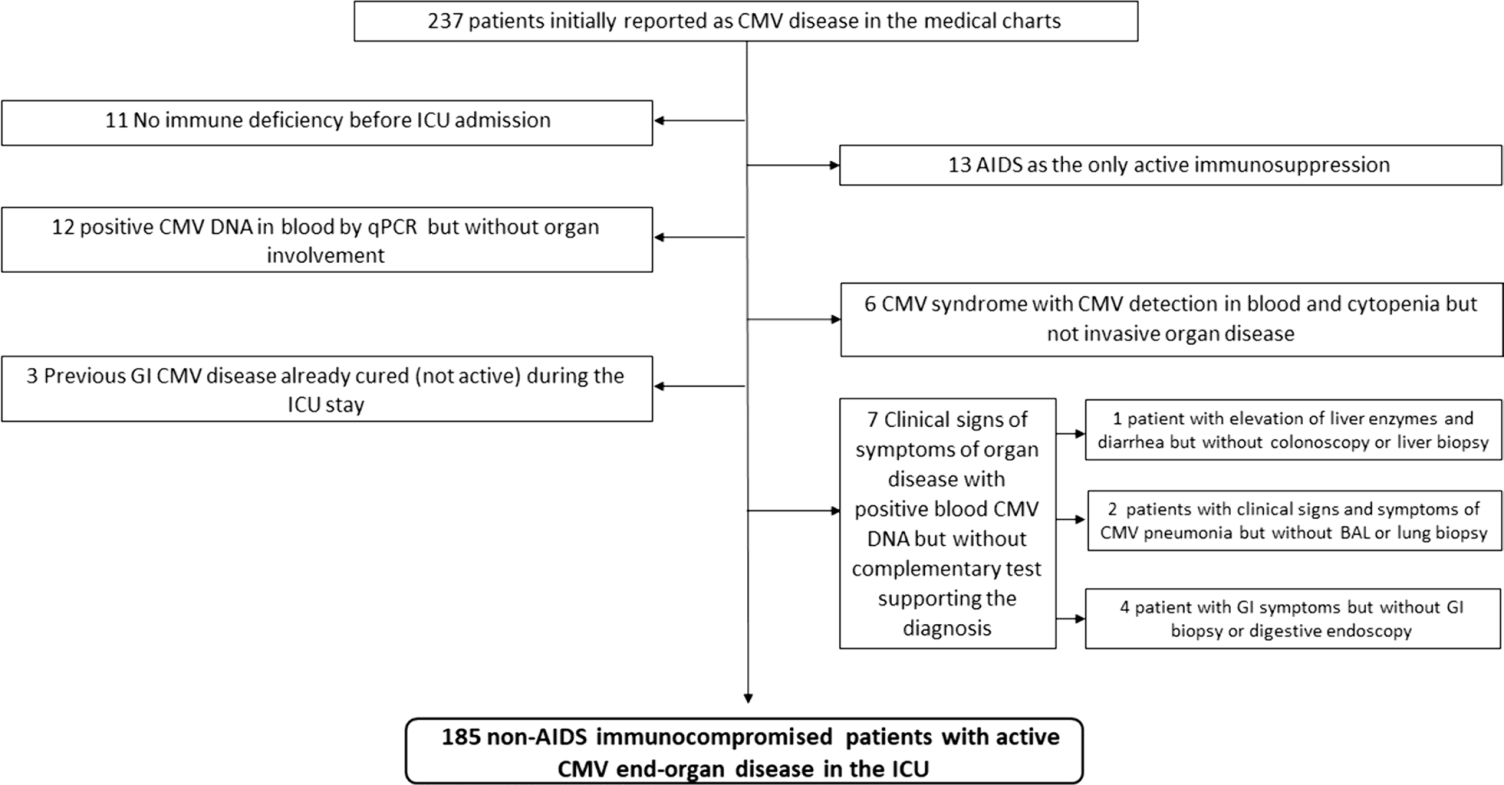
Patient flowchart. AIDS: Acquired immunodeficiency syndrome; BAL: Bronchoalveolar lavage; CMV: Cytomegalovirus; GI: Gastrointestinal; ICU: Intensive care unit; qPCR: Quantitative polymerase chain reaction

Figure 1 is the patient flowchart. Table 1 reports the main characteristics of the 185 included patients. Among them, 89 (48.1%) were not transplant recipients (HSCT or SOT) and 31 (16.8%) had immunosuppressant therapy as the only cause of immunodeficiency. Reason for ICU admission was related to CMV-EOD in half of the patients (53.5%). Patients who were discharged alive from hospital were follow up until day 90 after ICU admission. For more details on the underlying diseases among each type of immunodeficiency, see Additional file 1.

**Table 1:** Characteristics of the 185 study patients with CMV end-organ disease (CMV-EOD)

Variables		Median [IQR] or n (%)	Missing data
Age (years)		62 (53–69)	0
Males		114 (61.6)	0
HIV infection without AIDS^a^		4 (2.2)	0
Cause of immunosuppression			0
	Hematologic malignancy	80 (43.2)	
	Non-allogeneic HSCT^b^	47 (25.4)	
	Allogeneic HSCT without GVHD	12 (6.5)	
	Allogeneic HSCT with GVHD	21 (11.4)	
	Solid organ transplant	55 (29.7)	
	Drug-induced immunosuppression	31 (16.8)	
	Solid malignancy	16 (8.6)	
	Primary immunodeficiency	3 (1.6)	
Main reason for ICU admission			0
	Acute respiratory failure	103 (55.7)	
	Sepsis	32 (17.3)	
	Neurological symptoms	13 (7)	
	Hemorrhagic shock	10 (5.4)	
	Kidney or metabolic failure	10 (5.4)	
	Other	17 (9.2)	
SOFA score at ICU admission		6 (4–9)	5
Time of CMV-EOD diagnosis			0
	Before ICU admission	47 (25.4)	
	During the ICU stay	138 (74.6)	
Time from ICU admission and CMV-EOD diagnosis (days)^c^		3 (1–15)	0
CMV DNA in blood by qPCR			16
	Positive before ICU admission	89 (52.7)	
	Positive during the ICU stay	72 (42.6)	
	Negative	8 (4.3)	
Time from ICU admission to positive blood CMV qPCR^d^ (days)		6 (2–16)	0
Blood CMV viral load at diagnosis of CMV-EOD (IU/mL)		10 588 (1927–94 627)	16
Cytopenia at diagnosis of CMV end-organ disease			
	Leukopenia (< 4 × 10^9^/L)	54 (30.9)	10
	Lymphopenia (< 0.3 × 10^9^/L)	55 (34.4)	25
	Thrombocytopenia (< 150 × 10^9^/L)	114 (65.1)	10
Antiviral drugs used^e^			0
	Ganciclovir	161 (87)	
	Foscarnet	37 (20)	
	Valganciclovir	1 (0.5)	
	Cidofovir	2 (1.1)	
Organ support therapy during the ICU stay			0
	Vasopressors	115 (62.2)	
	HFNO or NIV	80 (45.5)	
	Invasive mechanical ventilation	126 (68.1)	
	Renal replacement therapy	57 (30.8)	
Length of ICU stay (days)		14 (6–31)	0
Length of hospital stay (days)		44 (27–70)	1
ICU mortality		75 (40.5)	0
Hospital mortality		113 (61.4)	1

^a^AIDS was a non-inclusion criterion, whereas patients with controlled HIV replication were eligible for inclusion

^b^Includes 8 patients with auto-HSCT

^c^considering only those patients who were diagnosed of CMV-EOD after ICU admission

^d^considering only those patients with active CMV replication in blood during the ICU stay with previous negative or unknown blood CMV qPCR (n = 72)

^e^Some patients received more than one antiviral drug alone or in combination during the ICU stay

*AIDS* Acquired immunodeficiency syndrome; *CMV* Cytomegalovirus; *CMV-EOD* Cytomegalovirus end-organ disease; *GVHD* Graft-versus-host disease; *HFNO* High-flow nasal oxygen; *HIV* Human immunodeficiency virus; *HSCT* Hematopoietic stem-cell transplantation; *ICU* Intensive care unit; *NIV* Non-invasive ventilation; *SOFA* Sequential Organ Failure Assessment

### Clinical presentations (Fig. 2)

**Fig. 2:**
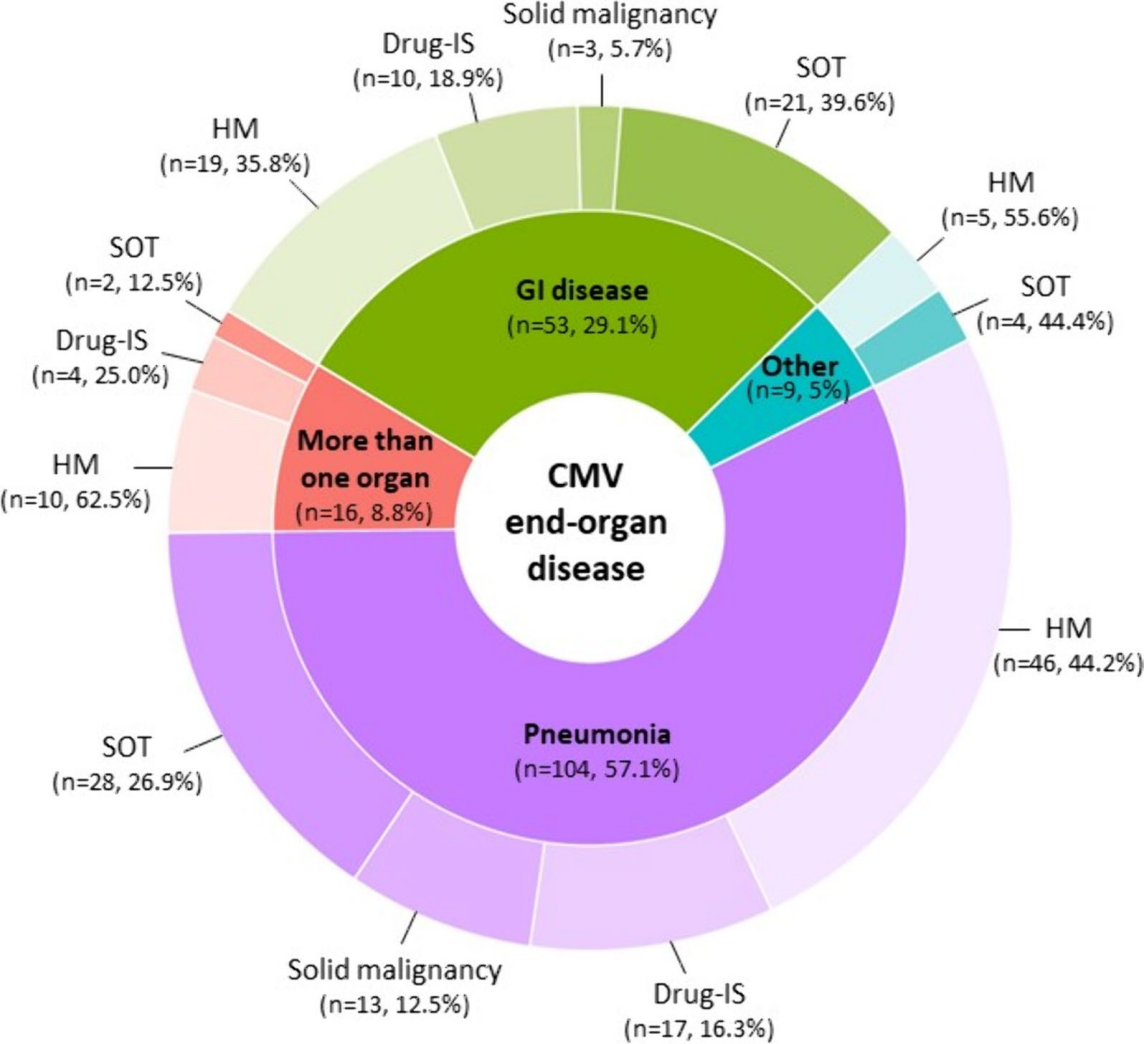
Types of CMV end-organ disease and underlying immune deficiencies. This graph does not include the 3 patients with primary immunodeficiency who had pneumonia, encephalitis, and gastrointestinal disease, respectively. The “other” category comprises 10 patients with retinitis (n = 4, 2.2%), hepatitis (n = 3, 1.6%), encephalitis (n = 2, 1.1%), or skin involvement (n = 1, 0.5%). Patients with more than one organ affected by CMV includes a combination of 10 pneumonia, 10 GI disease, 4 encephalitis, 3 retinitis, 5 hepatitis, 1 nephritis and 1 spleen involvement. CMV: Cytomegalovirus; Drug-IS: Drug-induced immunosuppression; GI: Gastrointestinal; HM: Hematologic malignancy; SOT: Solid organ transplant

Pneumonia was by far the most common CMV-EOD, followed by gastrointestinal (GI) involvement. All cases of encephalitis and 105 (91.3%) of the 115 cases of pneumonia were probable but not proven: symptoms and signs consistent with CMV disease were present and CMV tests on cerebrospinal fluid or bronchoalveolar lavage (BAL) fluid, respectively, were positive but histopathological samples were not obtained. Median CMV viral load in BAL fluid samples from patients with probable CMV pneumonia was 37 118 IU/mL (5599–337 235 IU/mL) while median CMV viral load in blood samples was significantly lower (1040 IU/mL [2064–51 070 IU/mL]; *p* < 0.001). When we compared CMV viral load in BAL and blood samples from patients with and without respiratory coinfection, no differences were found between the 2 groups (Additional file 2). All cases of CMV pneumonia in lung transplanted patients were diagnosed by histopathology. There were no differences in SOFA score at ICU admission between patients who presented CMV pneumonia and those who presented other CMV-EOD (*P* = 0.845). Median CMV viral load in CSF in patients with CMV encephalitis was 211 754 IU/mL (167 622–854 156 IU/mL) with no significant differences between levels of CMV viral load in blood (388 515 IU/mL [1593–3 2623 894]; *p* = 0.9). Of the 64 patients with GI disease, 43 (67.2%) had the diagnosis confirmed by histopathological examination of GI biopsies and the remaining 21 had positive qPCR results on GI tissue samples.

Of the 185 patients, 69 (37.3%) had other OIs during the ICU stay. The most common opportunistic pathogens were *Pneumocystis jirovecii* and *Aspergillus* sp. (n = 25, 13.5%, for each), followed by herpes simplex virus (n = 14, 7.6%)*.* Of the 115 patients with CMV pneumonia, 55 (47.8%) had recovery of a co-pathogen in the BAL fluid samples (Additional file 3).

### Outcomes

**Fig. 3:**
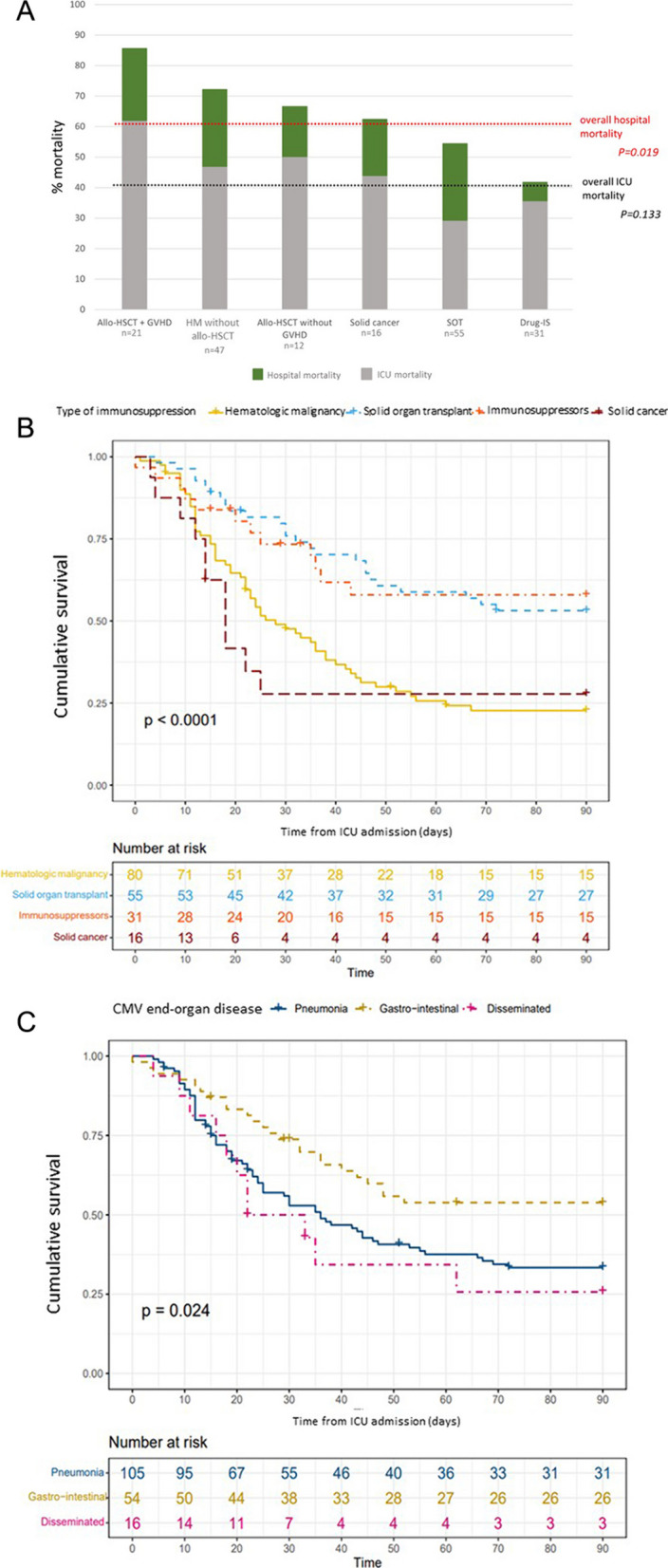
Mortality rates and cumulative survival curves. **A** Hospital mortality according to the cause of immunodeficiency. All 3 patients with primary immunosuppression were discharged alive from the hospital. Mortality rates were compared using the chi-square test. **B** Cumulative overall day-90 survival according to the cause of immunodeficiency. **C** Cumulative overall day-90 survival according to the type of CMV end-organ disease. Survival was plotted according to the Kaplan–Meier method and comparisons were with the log-rank test. In panels B and C, 15 (8.1%) patients were lost to follow-up. Allo-HSCT: allogeneic hematopoietic stem-cell transplant; Drug-IS: drug-induced immunosuppression; GVHD: graft-versus-host disease; ICU: intensive care unit; SOT: solid organ transplant

Of the 185 patients, 113 (61.1%) died in the hospital, including 75 (75/113, 66.4%) in the ICU. Hospital mortality was higher in the group with hematologic malignancies than in the groups with other causes of immunodeficiency. The highest hospital mortality rate was in allogeneic HSCT recipients with active GVHD, of whom 85.7% (19/21) died, usually in the ICU (Fig. 3A). Day-90 mortality was lowest in the groups with SOT or drug-induced immunosuppression (Fig. 3B).

Patients with CMV pneumonia or CMV involvement of more than one organ had significantly higher hospital mortality (72/105, 69.2% and 10/16, 62.5%, respectively) than did patients with GI disease (25/54 46.3%) (Fig. 3C). Within the CMV-pneumonia group, day-90 mortality was not significantly different between patients with vs. without co-infection at the time of diagnosis (Additional file 4).

CMV treatment was changed in 17 patients (9.2%): 8 (8/17, 47.1%) due to secondary cytopenia, 2 (2/17, 11.8%) due to renal failure and 7 (7/17, 41.2%) due to lack of response to treatment.

### Risk factors for hospital mortality

**Fig. 4:**
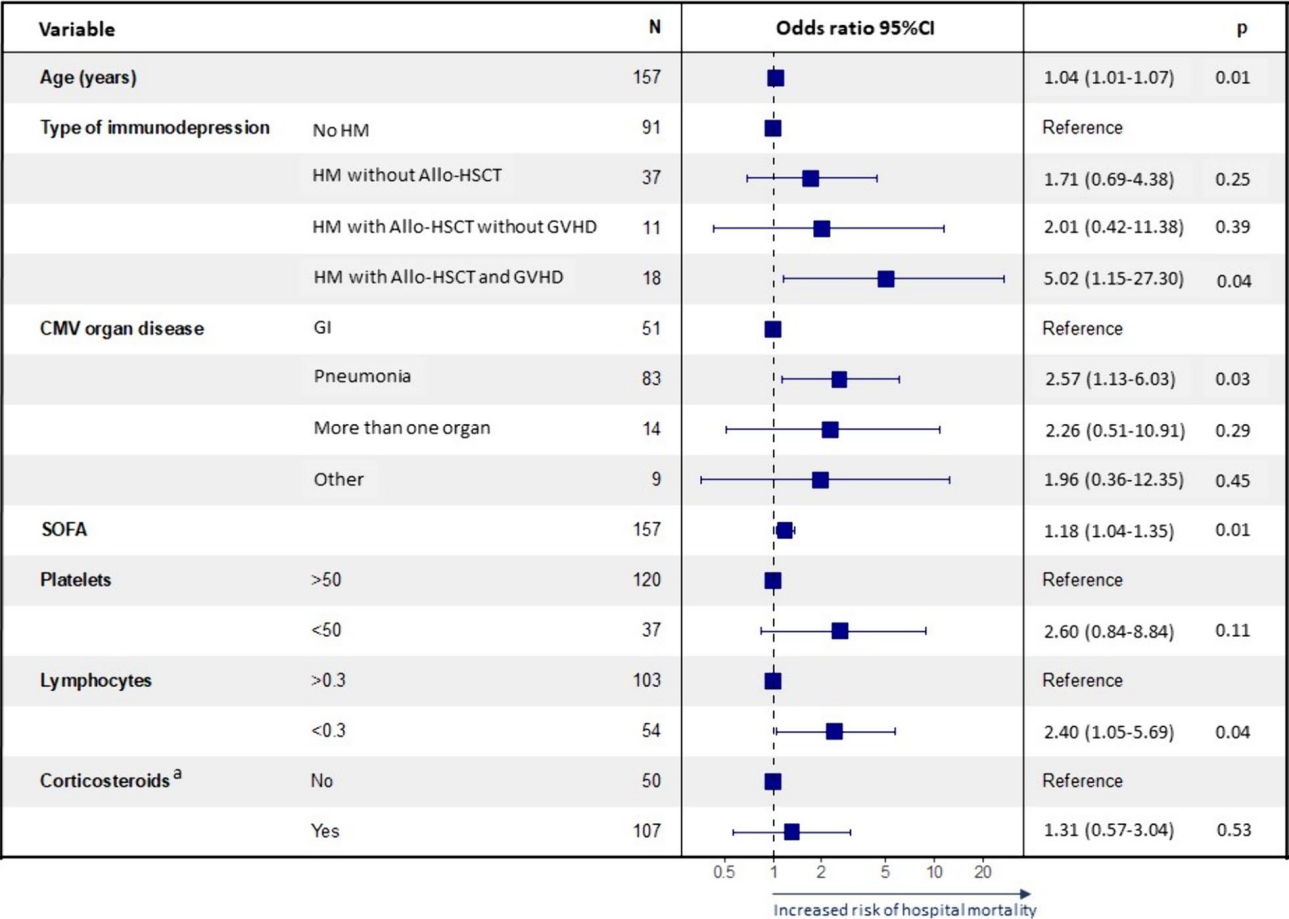
Factors independently associated with hospital mortality by logistic regression. 95% CI 95% confidence interval; Allo-HSCT: Allogeneic hematopoietic stem-cell transplantation; CMV: Cytomegalovirus; GI: Gastrointestinal; GVHD: Graft-versus-host disease; HM: Hematologic malignancy; SOFA: Sequential organ failure assessment. ^a^Patients on corticosteroid therapy at ICU admission that includes high-dose (> 0.5 mg/kg/day) or long-term (> 30 days) for patients without any other immunosuppressive condition and any dose within the last 30 days before ICU admission for the other groups of patients with another cause of immunosuppression

Table 2 reports the comparison of survivors and non-survivors at hospital discharge. In-hospital death was associated with hematologic malignancy; severe lymphopenia (< 0.3 × 10^9^/L); severe thrombocytopenia (50 × 10^9^/L); experiencing another OI during the ICU stay, particularly aspergillosis; and needing a higher number of organ-support therapies. Neither blood CMV load at diagnosis nor previous corticosteroid therapy were associated with hospital mortality. By multivariate analysis adjusted for confounders, hematologic malignancy with active GVHD, CMV pneumonia, lymphocyte count below 0.30 × 10^9^/L at diagnosis of CMV-EOD, worse SOFA score at ICU admission, and older age were independently associated with hospital mortality (Fig. 4).

**Table 2:** Comparison of clinical characteristics in hospital survivors and non-survivors

Variables Median [IQR] or n (%)			Survivors n = 71	Non survivors n = 113	*P* value^a^
Age (years)			62 (49–68)	63 (55–69)	0.086
Males			41 (57.7)	72 (63.7)	0.492
Cause of immunosuppression					
	Hematologic malignancy		20 (28.2)	60 (53.1)	0.001
		Non-allogeneic HSCT	13 (18.3)	34 (30.1)	0.084
		Allogeneic HSCT without GVHD	4 (5.6)	8 (7.1)	0.769
		Allogeneic HSCT with GVHD	3 (4.2)	18 (15.9)	0.017
	Solid organ transplant		24 (33.8)	30 (26.5)	0.321
	Drug–immunosuppression		18 (25.4)	13 (11.5)	0.025
	Solid cancer		6 (8.5)	10 (8.8)	1
	Primary immunodeficiency		3 (4.2)	0	0.056
Corticosteroid therapy^b^			46 (64.8)	84 (74.3)	0.186
SOFA score at ICU admission			5 (4–7)	7 (5–10)	< 0.001
Lymphocytes < 0.30 × 10^9^/L			14 (21.2)	41 (44.1)	0.004
Platelets < 50 × 10^9^/L			6 (8.7)	37 (35.2)	< 0.001
Blood CMV load at diagnosis (IU/mL)			9940 (1121–60 106)	10 936 (2614–136 368)	0.281
CMV end-organ disease					
	Pneumonia		36 (50.7)	78 (69)	0.019
	Gastrointestinal tract		33 (46.5)	31 (27.4)	0.011
	More than one organ		6 (8.5)	10 (8.8)	1
Other infections during the ICU stay			50 (70.4)	92 (81.4)	0.086
Respiratory co-infection^c^			12 (33.3)	41 (52.6)	0.070
Other opportunistic infection during the ICU stay			18 (25.4)	51 (45.1)	0.008
Aspergillosis during the ICU stay			3 (4.2)	22 (19.5)	0.003
Organ support therapy during the ICU stay					
	Vasopressors		31 (43.7)	83 (73.5)	< 0.001
	Invasive mechanical ventilation		35 (49.3)	90 (79.6)	< 0.001
	Renal replacement therapy		13 (18.3)	43 (38.1)	0.005

^a^Categorical variables were compared using Fisher’s exact test and continuous variables using the Mann–Whitney test

^b^Patients on corticosteroid therapy at ICU admission that includes high-dose (> 0.5 mg/kg/day) or long-term (> 30 days) for patients without any other immunosuppressive condition and any dose within the last 30 days before ICU admission for the other groups of patients with another cause of immunosuppression

^C^Patients with CMV pneumonia

*CMV* Cytomegalovirus; *HSCT* Hematopoietic stem-cell transplantation; *GVHD* Graft-versus-host disease; *ICU* Intensive care unit; *SOFA* Sequential organ failure assessment

## Discussion

CMV-EOD among critically ill, immunocompromised patients was shown in this study to have multiple clinical presentations and to occur in patients with a variety of underlying immunodeficiencies. Among these, the most common were hematologic malignancies, more than half with HSCT and notably with active GVHD, and SOT. CMV pneumonia, the most often life-threatening form of CMV-EOD, was the most common presentation. Two-fifths of patients died in the ICU and nearly two-thirds before hospital discharge.

The incidence of CMV-EOD in high-risk transplant recipients has decreased since the introduction nearly three decades ago of prophylactic and preemptive antiviral treatment strategies. In a 2016 study of allogeneic HSCT recipients who were CMV-seropositive and received CMV-seropositive transplants, 95/926 (10.3%) patients developed CMV-EOD [17] However, CMV-EOD caused only 1% of the 263 deaths that occurred within the first year. Of 1239 patients given solid organ transplants in 2008–2011, 75 (6.1%) experienced CMV-EOD, which was not significantly associated with graft loss or death [18]. These studies were not performed in the setting of critical illness, which may increase the risk of CMV reactivation progressing to CMV-EOD.

CMV-EOD may be the reason for ICU admission if responsible for severe organ dysfunction or may develop during the ICU stay, as critical illness increases the risk of CMV reactivation. Among immunocompetent ICU patients who were seropositive for CMV, up to a third developed CMV reactivation manifesting as a rise in CMV loads over time [19–21]. CMV reactivation during critical illness was associated with higher mortality in three studies [5, 19, 20] but not in another [21]. All these studies were done in immunocompetent patients and focused on CMV reactivation as opposed to CMV-EOD. In transplant patients without critical illness, the risk of CMV-EOD was greater in patients with higher viral loads early during reactivation and with a faster viral load increase over time [9]. Thus, serial qPCR testing to monitor viral load changes may assist in the early detection of CMV-EOD in critically ill, immunocompromised patients. Guidelines issued in 2019 for patients with hematologic malignancies recommend routine qPCR monitoring [13]. Of our 185 patients, 72 (42.6%) had negative qPCR tests before ICU admission and experienced CMV reactivation during the ICU stay, a median of 6 days after admission. Median blood CMV DNA levels in these patients were 14 480 IU/mL (3620-61 243 IU/mL). Of note, 8 (4.3%) patients had a negative qPCR blood test for CMV at the time CMV-EOD was diagnosed, confirming that local virus replication can occur without systemic involvement [22, 23]. One of these patients had CMV retinitis, confirmed by an experienced ophthalmologist. Four presented GI disease: 50% with histological confirmation in tissue samples and 50% with compatible macroscopic mucosal lesions in endoscopy and high qPCR in tissue samples (47 583 IU/mL and 76 080 IU/mL) with negative viremia and without any other alternative diagnosis, therefore considered as a possible GI disease. And 3 were diagnosed as probable CMV pneumonia due to the presence of respiratory symptoms and compatible radiological findings, along with a positive qPCR in BAL fluid (11 065 IU/mL, 3433 IU/mL and 21 532 IU/mL), a negative viremia and no other coexisting respiratory infection.

Although the contribution of CMV-EOD on clinical outcomes such as the need for organ support or mortality is difficult to assess due to the frequent coexistence with other pathologies, CMV-EOD may reflect the vulnerability and degree of T-cell deficiency of these patients, with high rates of associated mortality. In our study, mortality was highest in allogeneic HSCT recipients. In this population, critical illness is often fatal even in the absence of CMV infection, particularly when acute GVHD develops [24]. The risk of CMV replication is increased by acute GVHD and vice versa [25]. Among our patients with solid malignancies, over half had metastatic disease, nearly nine-tenths had received chemotherapy, and a third were on corticosteroid therapy. All these factors would be expected to increase the risk of death.

Pneumonia, a severe form of CMV-EOD, was present in over half our patients. The 69.2% mortality rate in patients with CMV pneumonia is consistent with earlier data [26]. CMV pneumonia is more common in allogeneic HSCT and lung transplant recipients comparing to other type of immunocompromised patients, in whom rates between 25 and 30% [27, 28] have been described. The incidence of CMV pneumonia in our cohort represents 57.1% of all patients, which is higher than those previously described in the literature. These differences may be explained because most studies on prevalence of CMV-EOD in the literature are performed outside the ICU. Our results reflect the severity of CMV pneumonia, which can induce organ dysfunction and ICU admission more often than other CMV-EOD. Additionally, respiratory distress syndrome from another cause may lead to CMV lung reactivation in critically ill immunocompromised patients, increasing the risk of developing CMV pneumonia. GI disease was the second most common CMV organ involvement. In a retrospective study, a third of patients with hematologic or solid malignancies and GI CMV disease required ICU admission [29]. Malnutrition and sepsis, two common conditions in critically ill, immunocompromised patients, were associated with higher mortality in patients with GI CMV disease [30].

Cytopenia was common in our patients. CMV infection may induce myelosuppression via marrow-cell infection and/or indirect immune-mediated effects [31, 32]. A lymphocyte count below 0.3 × 10^9^/L was an independent risk factor for mortality, as expected given the importance of cellular immunity in suppressing CMV replication.

OIs other than CMV infection occurred in over a third of our patients. Among patients with CMV pneumonia, 24/115 (20.9%) also had aspergillosis and 23/115 (20%) had *P. jirovecii* pneumonia. Several studies found that CMV-EOD was associated with an increased frequency of invasive fungal disease in HSCT and SOT recipients [33].

A major limitation of our study is the retrospective design, which is inevitably associated with information bias. Second, most cases of CMV pneumonia were not confirmed by histopathological evidence but were instead probable diagnoses based on qPCR loads, viral culture or presence of viral inclusions in BAL fluid. Optimal cutoffs for defining positive qPCR testing on BAL fluid have not been established and may vary according to the cause of the immunodeficiency [34]. Thus, overdiagnosis may have occurred, with CMV shedding being mistaken for active CMV replication. Moreover, 47% of patients with CMV pneumonia had a co-pathogen isolated from BAL fluid at the time of CMV pneumonia diagnosis. The specific role for each pathogen in the symptoms and outcomes cannot be determined. Third, as indicated previously, the specific contribution of CMV-EOD to the high mortality cannot be estimated from our data. Our study nonetheless provides valuable information, as it is the first to assess CMV-EOD, defined according to Ljungman et al. [35], in critically ill, immunocompromised patients and to show the distribution of the different organ affected by CMV among the different types of underlying immunosuppression. The little literature that exists on CMV disease in immunocompromised patients focuses only on specific populations such as those with hematological malignancies [36] or after solid organ transplant [37], mainly on CMV reactivation and with very broad defining criteria for CMV disease. Also, we compared outcomes according to the cause of immunodeficiency. Finally, the multicenter international recruitment supports the external validity of our findings.

## Conclusions

Critically ill, immunocompromised patients with CMV-EOD vary widely regarding the cause of immunodeficiency and the organ involved. Hematologic malignancy was the most common underlying disease of which, more than half were HSCT recipients and pneumonia the most common manifestation of CMV-EOD. Other OIs were often present. Mortality was high, notably in HSCT recipients with active GVHD. The contribution of CMV-EOD to this high mortality is unclear. Further work is needed to determine the optimal diagnostic and treatment strategies for CMV reactivation and end-organ involvement in this population.

## Supplementary Information

Additional file 1 Underlying diseases among each type of immunodeficiency.

Additional file 2 CMV viral load in BAL fluid and blood samples from patients with probable CMV pneumonia.

Additional file 3 Opportunistic infections during the ICU stay. HSV: herpes simplex virus; HHV6: human herpes virus 6; VZV: varicella-zoster virus.

Additional file 4 Data on patients with CMV pneumonia and co-pathogens. (A) Co-pathogens isolated from respiratory specimens at same time as CMV. (B) Cumulative overall day-90 survival in patients who had CMV pneumonia with vs. without a co-pathogen. Kaplan-Meier survival curves and comparisons using the log-rank test. In panel B, 15 patients (8.1%) were lost to follow-up.

## Data Availability

The datasets used and/or analyzed during the current study cannot be made publicly available due to data privacy regulations but are available from the corresponding author on reasonable request.

## Abbreviations

AIDS: Acquired immunodeficiency syndrome
BAL: Bronchoalveolar lavage
CMV: Cytomegalovirus
CMV-EOD: Cytomegalovirus end-organ disease
GI: Gastrointestinal
GVHD: Graft-versus-host disease
HSCT: Hematopoietic stem-cell transplantation
ICU: Intensive care unit
OI: Opportunistic infection
qPCR: Quantitative polymerase chain reaction
SOFA: Sequential Organ Failure Assessment
SOT: Solid organ transplantation
