# Supplementary material for: Clinical characteristics and outcomes of immunocompromised critically ill patients with cytomegalovirus end-organ disease: a multicenter retrospective cohort study
Source: Crit Care. 2024 Jul 16;28:243. doi: 10.1186/s13054-024-05029-4 (PMC11251242; doi:10.1186/s13054-024-05029-4)
Supplement: Supplementary file 1 — Additional file 1 Underlying diseases among each type of immunodeficiency. [file 13054_2024_5029_MOESM1_ESM.docx]

| **Patients with hematologic malignancy (n=80)** | | | | | |
| --- | --- | --- | --- | --- | --- |
| **Type of hematologic malignancy** | | | **Hematopoietic stem cell transplantation (HSCT)** | | |
| Non-Hodgkin lymphoma | | 24 (30%) | Allo-HSCT | | 33 (41.2%) |
| Multiple myeloma | | 9 (11.2%) | Auto-HSCT | | 8 (10%) |
| Acute myeloid leukemia | | 8 (10%) | No HSCT | | 39 (48.8%) |
| Myelodysplastic syndrome | | 8 (10%) | Active GVHD at ICU admission | | |
| Acute lymphoid leukemia | | 7 (8.8%) |  | Grade 1-2 | 14(17.5%) |
| T-cell lymphoma | | 7 (8.8%) |  | Grade 3-4 | 7 (8.8%) |
| Myeloproliferative syndrome | | 5 (6.2%) | Time between HSCT and ICU admission | | |
| Chronic lymphoid leukemia | | 3 (3.8%) |  | <30 days | 9 (11.2%) |
| Hodgkin lymphoma | | 2 (2.5%) |  | 30-100 days | 10 (12.5%) |
| Others | | 7 (8.8%) |  | >100 days | 22 (27%) |
| **Patients with solid organ transplant (n=55)** | | | | | |
| **Type of SOT** | | | **Time between SOT and ICU admission** | | |
| Kidney | | 25 (49.1%) | <30 days | | 17 (30.9%) |
| Liver | | 18 (32.7%) | 30-100 days | | 4 (7.3%) |
| Lung | | 5 (9.1%) | >100 days | | 34 (61.8%) |
| Heart | | 4 (7.3%) |  | | |
| Kidney-heart | | 1 (1.8%) |  |  |  |
| **Patients with drug-induced immunosuppression (n=31)** | | | | | |
| **Type of underlying disease** | | | **Immunosuppressive treatments** | | |
| Bowel inflammatory disease | | 7 (22.6%) | Corticosteroids | | 25 (80.5%) |
| Connective tissue/rheumatologic disease | | 5 (16.1%) | Azathioprine | | 10 (32.3%) |
| Vasculitis | | 4 (12.9%) | Mycophenolate | | 5 (16%) |
| Myasthenia gravis | | 3 (9.7%) | Rituximab | | 4 (12.9%) |
| Non-malignant hematologic disease | | 3 (9.7%) | Cyclophosphamide | | 3 (9.7%) |
| Other systemic immune disease | | 5 (16.1%) | Methotrexate | | 3 (9.7%) |
| Others | | 4 (12.9%) |  | | |
| **Patients with solid malignancy (n=16)** | | | | | |
| Metastatic cancer | | | | | 9 (56.2%) |
| Cancer therapy | | | | |  |
|  | Chemotherapy | | | | 14 (87.5%) |
|  | Radiotherapy | | | | 7 (43.8%) |
|  | Corticosteroids | | | | 5 (31.2%) |
|  | Target directed therapy | | | | 1 (6.2%) |
|  | Immunotherapy | | | | 3 (18.8%) |
| **Patients with primary immunodeficiency (n=3)** | | | | | |
| Common variable immunodeficiency (CVID) | | | | | 1 (33.3%) |
| Idiopathic CD4 lymphopenia | | | | | 1 (33.3%) |
| IFN γ-secretion deficit | | | | | 1 (33.3%) |

**Additional file 1. Underlying diseases among each type of immunodeficiency**

All variables are expressed as absolute number and percentage. Allo-HSCT: allogeneic hematopoietic stem cell transplantation; Auto-HSCT: autologous hematopoietic stem cell transplantation; GVHD: graft-versus-host disease; ICU: intensive care unit; SOT: solid organ transplant; IFN: interferon.
