# Supplementary material for: Clinical characteristics and outcomes of immunocompromised critically ill patients with cytomegalovirus end-organ disease: a multicenter retrospective cohort study
Source: Crit Care. 2024 Jul 16;28:243. doi: 10.1186/s13054-024-05029-4 (PMC11251242; doi:10.1186/s13054-024-05029-4)
Supplement: Supplementary file 2 — Additional file 2 CMV viral load in BAL fluid and blood samples from patients with probable CMV pneumonia. [file 13054_2024_5029_MOESM2_ESM.docx]

|  | **Probable CMV pneumonia** | | | |
| --- | --- | --- | --- | --- |
|  | **All episodes of probable pneumonia**  **n=105** | **Without respiratory coinfection**  **(n=58)** | **With respiratory coinfection**  **(n=47)** | ***P* value^b^** |
| **Median CMV DNA in BAL fluid (IQR) IU/mL** | 37 118 (5599 – 337 235) | 42 343 (5 571-225 034) | 28 330 (9 585- 465 384) | 0.839 |
| **Median CMV DNA in blood (IQR) IU/mL** | 10 040 (2064 – 51 070) | 9665 (2221 – 38 258) | 10 893 (1965 – 93 437) | 0.541 |
| ***P* value^c^** | P < 0.001 | P= 0.009 | P=0.005 |  |

**Additional file 2. CMV viral load in BAL fluid and blood samples from patients with probable CMV pneumonia^a^**

^a^Patients with proven CMV pneumonia (n=10) were excluded from this analysis.

^b^Comparison between probable CMV pneumonia with and without a co-pathogen isolated from BAL fluid at time of CMV pneumonia diagnosis using the Mann-Whitney test.

^c^Comparison between median CMV viral load levels in BAL fluid and blood samples in each group using the Wilcoxon signed-rank test.

CMV: cytomegalovirus; BAL: bronchoalveolar lavage; IQR: interquartile range.
