# Supplementary figures and images for: Clinical characteristics and outcomes of immunocompromised critically ill patients with cytomegalovirus end-organ disease: a multicenter retrospective cohort study
Source: Crit Care. 2024 Jul 16;28:243. doi: 10.1186/s13054-024-05029-4 (PMC11251242; doi:10.1186/s13054-024-05029-4)

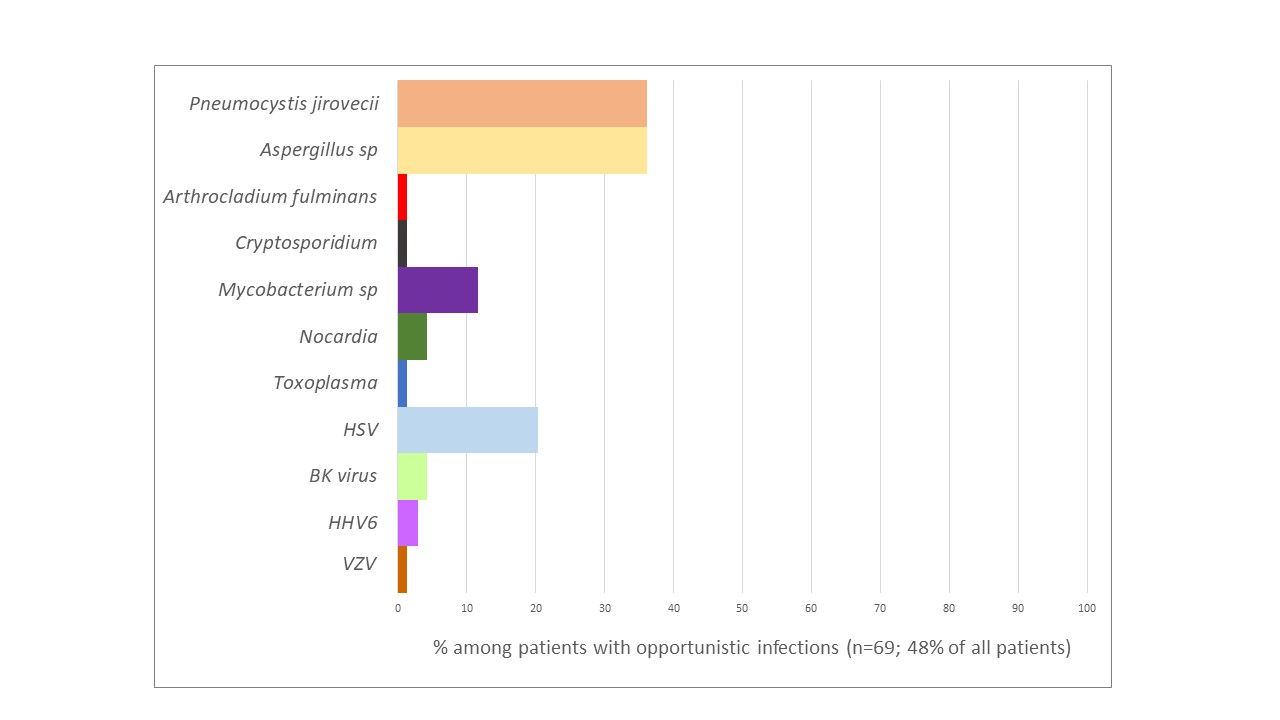

Supplement: Supplementary file 3 — Additional file 3 Opportunistic infections during the ICU stay. HSV: herpes simplex virus; HHV6: human herpes virus 6; VZV: varicella-zoster virus. [file 13054_2024_5029_MOESM3_ESM.jpg]

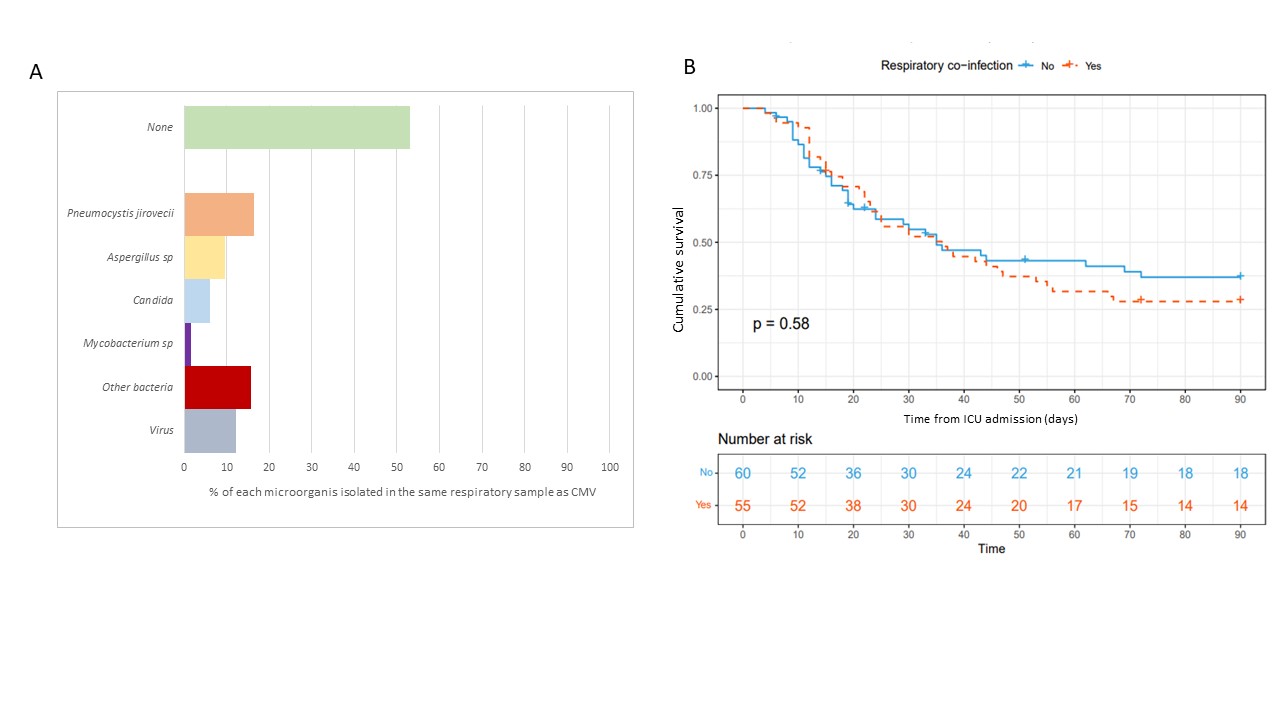

Supplement: Supplementary file 4 — Additional file 4 Data on patients with CMV pneumonia and co-pathogens. (A) Co-pathogens isolated from respiratory specimens at same time as CMV. (B) Cumulative overall day-90 survival in patients who had CMV pneumonia with vs. without a co-pathogen. Kaplan-Meier survival curves and comparisons using the log-rank test. In panel B, 15 patients (8.1%) were lost to follow-up. [file 13054_2024_5029_MOESM4_ESM.jpg]
